# Ambient PM_2.5_ exposures could increase risk of tuberculosis recurrence

**DOI:** 10.1265/ehpm.23-00131

**Published:** 2023-08-31

**Authors:** Kyung-Duk Min, Sun-Young Kim, Sung-il Cho

**Affiliations:** 1 College of Veterinary Medicine, Chungbuk National University; 2 Department of Cancer Control and Population Health, Graduate School of Cancer Science and Policy, National Cancer Center; 3 Department of Public Health Sciences, Graduate School of Public Health, Seoul National University; 4 Institute of Health and Environment, Graduate School of Public Health, Seoul National University

**Keywords:** Tuberculosis, Particulate matter, Spatial epidemiology

## Abstract

**Background:**

The effect of ambient PM_2.5_ on the incidence of tuberculosis (TB) has been investigated in epidemiological studies. However, they did not separately study new and relapsed TB infection and focused on relatively short-term effects of PM_2.5_. In this regard, we examined the associations of long-term PM_2.5_ exposures with both new and relapsed TB incidences in South Korea, where the disease burden of TB is greatest among high-income countries.

**Methods:**

An area-level ecological study of 250 districts was conducted from 2015 to 2019. Age- and sex-standardized TB incidence ratios for each district and year were used as outcome variables, and their associations with PM_2.5_ concentrations for one to five-year average were examined. Negative binomial regression models incorporating spatiotemporal autocorrelation were employed using integrated nested Laplace approximations. Stratified analyses were conducted by type of TB (total, new, and relapsed cases).

**Results:**

Districts with higher PM_2.5_ concentrations tended to have significantly higher TB recurrence rate. The relative risks per 10 µg/m^3^ PM_2.5_ increase were 1.218 (95% credible interval 1.051–1.411), 1.260 (1.039–1.527) and 1.473 (1.015–2.137) using the two, three and five-year average PM_2.5_ exposures, respectively.

**Conclusions:**

The results imply that interventions for reducing air pollution might help prevent TB recurrence.

**Supplementary information:**

The online version contains supplementary material available at https://doi.org/10.1265/ehpm.23-00131.

## Background

Tuberculosis (TB) is caused by *Mycobacterium tuberculosis* and is transmitted from infectious individuals via air or droplets. The global disease burden of chronic TB infection is high, with approximately 10 million newly reported patients and 1.2 million deaths in 2019 [1]. While the disease burden of TB tends to be higher in lower- or middle-income countries, South Korea had 23,821 cases (388 per million) and approximately 1,610 TB-related deaths in 2019 [2], which is the highest burden among Organization for Economic Cooperation and Development (OECD) countries. The prevalence of latent TB is also high, with an estimate of 33.2% in 2016 [3].

There are various risk factors for TB infections, including biomedical, health behavior, and socioeconomic factors. In a literature review, Silva et al. suggested that diabetes mellitus, smoking, alcohol use, and illicit drug use increased the susceptibility to TB infection [4]. In a narrative review, Lonnroth et al. stated that low socioeconomic status (SES) increases the risk of TB infection because people with a low SES tend to be undernourished, ignore personal hygiene, and have poor ventilation in their home and work places [5]. The national SES could affect the risk. The TB disease burden tends to be higher in low- and middle-income countries [1]. In South Korea, the incidence of TB was high during the Korean War but decreased as the gross domestic product increased [6]. The direct and indirect effects of SES on the morbidity and mortality of infectious diseases have been well-described elsewhere [7].

Particulate matter that have a diameter of less than 2.5 micrometers (PM_2.5_) have also been suggested as a possible risk factor based on biological plausibility. Gonzalez et al. found decreased production of TNF-α in alveolar macrophages after exposure to PM_2.5_ [8, 9]. Considering the roles of TNF-α in TB, including macrophage activation, cell recruitment to infection sites, and granuloma formulation [10], exposure to PM_2.5_ could increase the risks of both initial infection and reactivation of TB.

Several epidemiological studies also supported the association between ambient PM_2.5_ exposures and TB incidence. You et al. found that a higher winter PM_2.5_ concentration resulted in higher TB incidences in the following spring and summer in Beijing and Hong Kong [11]. Studies with time-series analysis revealed higher level of PM_2.5_ was associated with higher number of TB cases in Shanghai [12] and Jinan, China [13]. Studies in the USA also reported the positive associations [14, 15]. However, those studies used all TB incidence cases regardless of whether it is new infection or recurrence as an outcome variable. Because main causes for the two types of TB cases are different, i.e., the major cause of new TB infection is primary exposure to active TB patients but that of relapse cases is reactivation [16], the effect of PM_2.5_ could be different by the type of TB. In addition, the previous studies tended to focus on short-term effects of PM_2.5_ exposures (less than a few months). The potential long-term effects of PM_2.5_ exposures are valuable to be investigated as other health outcomes [17–19].

In order to address the current research gaps, we investigated associations between long-term exposures to ambient PM_2.5_ and both new and recurrent TB incidence in South Korea where the burden of TB is highest among OECD countries and the effect of PM_2.5_ exposures has not been studied. Although two recent South Korean studies investigated the associations of TB incidence with criteria air pollutants including PM_10_, CO, O_3_, and NO_2_ [20, 21], PM_2.5_ was not included in those studies possibly because nationwide PM_2.5_ monitoring data became available recently (after 2015).

## Methods

A cross-sectional ecological study design was used to assess the effect of PM_2.5_ on TB incidence across the 250 districts in South Korea. The sex- and age-standardized incidence ratio (SIR) for annual new TB (new TB SIR), relapsed TB (relapsed TB SIR), and all TB (all TB SIR) were the outcome variables and PM_2.5_ concentrations for one to five-year average were the exposure variables. Because we used both TB and PM_2.5_ concentrations between 2015 and 2019, the number of subjects is different by the time window. For instance, TB outcome data in 2015 was not used in the analysis using two-year time period for PM_2.5_ exposures (N = 1,000) and TB outcome data only in 2019 was used in the analysis using five-year time period for PM_2.5_ exposures (N = 250). TB-related health behaviors (proportions of smokers [4] and binge drinkers [4]) and co-morbidity (obesity [22] and diabetes [4] prevalence) were included as covariates. Quality of life index and budget dependency were also included as SES variables [5] which could be related to TB risks. Population density was another covariate because risk of contacts to active TB individuals could be higher in the regions with higher population density. Variables were acquired for each year of the study period from 2015 to 2019.

In this study, annual mean PM_2.5_ concentrations were predicted in each district each year from 2015 to 2019 to assess the long-term ambient exposures. We used predicted PM_2.5_ instead of measurements, because there are no air pollution monitoring stations in approximately 40% of districts [23]. In South Korea, the Ministry of Environment have been operated 294 regulatory monitoring sites for air pollution and the hourly measurement data for PM_2.5_ were publicly available. PMs at sites are measured using a beta attenuation monitoring (BAM) technique [24]. Daily average value was computed from the hourly data only in case there were more than 18 hourly measurement. Among the 294 sites, we used measurements from 277 sites which met our inclusion criteria for computing annual average concentration; 1) average PM_2.5_ concentration level at least for 9 months should be available, 2) daily average concentration should not be missing consecutively for 45 days. Subsequently, we estimated the annual mean PM_2.5_ concentrations at 83,463 centroids of residential census tracts (mean area of census tracts is 1.2 km^2^) using exposure prediction models. Universal kriging method was employed in the prediction model using regulatory monitoring data of PM_2.5_ at 277 sites and more than 300 geographic variables related to potential pollution sources, including traffic, demographic characteristics, land use, physical geography, transportation facilities, emissions, vegetation, and altitude [25]. Subsequently, the predicted annual mean PM_2.5_ concentrations at census tract centroids were averaged to each district.

The Korea Disease Control and Prevention Agency (KDCA) provides the number of new and relapse TB cases by sex and age groups and by districts annually [26]. Using the reported number of TB cases by sex and age groups and population size of each group in 2015, incidence rates of each group were calculated. Using the incidence rates and population size by each district and year, expected number of new and relapse TB cases were calculated for each district in 2015–2019. Subsequently, the sex- and age-standardized annual TB incidence ratio for each district in 2015–2019 were calculated by the ratio of reported and expected number of cases. In terms of the covariates, district-level data for each year were obtained from the Korean Statistical Information Service (KOSIS) [27]. EuroQol-5D index was used for the district level quality of life which measures five health-related dimensions: mobility, self-care, usual activities, pain/discomfort and anxiety/depression [28]. Budget dependency indicates proportion of sum of local tax revenue and non-tax revenue to total revenue for each district implying socio-economic levels. Those who have smoked more than 100 cigarettes in their lifetime and are still smokers were defined as smokers. People who drink more than the standard alcohol limits twice a week were defined as binge drinkers. The standard alcohol limit is five cans of beer for males and three cans of beer for females. Obesity is defined as body mass index (kg/m^2^) higher than or equal to 25. Those who are older than 30 and have diagnosed diabetes are defined as diabetes patients.

Descriptive analyses were implemented to show the differences in PM_2.5_ concentration and covariates between districts with higher total TB SIR (higher than median value) and the other districts from 2015 to 2019. Choropleth maps with quintile categorization were provided to show the regional distributions of the variables.

Poisson and negative binomial (NB) models were used to examine the associations of the district-level PM_2.5_ concentrations with all, new, and relapsed TB SIR. Reported number of cases were used as response variables and the expected number of cases were used as an offset in the models. The goodness of fit of the both Poisson and NB models was examined using the deviance information criterion [29]; the model with the lowest deviance information criterion (DIC) was selected as the best-fit model. Considering possible autocorrelations by both time (year) and space, hierarchical approaches were incorporated [30]. For Bayesian inference, integrated nested Laplace approximation (INLA) approaches in the INLA package [31] in R v. 3.5.1 [32] were used. The results for all models are presented as relative risks (RRs) per 10 µg/m^3^ PM_2.5_ increase and 95% credible intervals (95% CrIs).

## Results

**Fig. 1:**
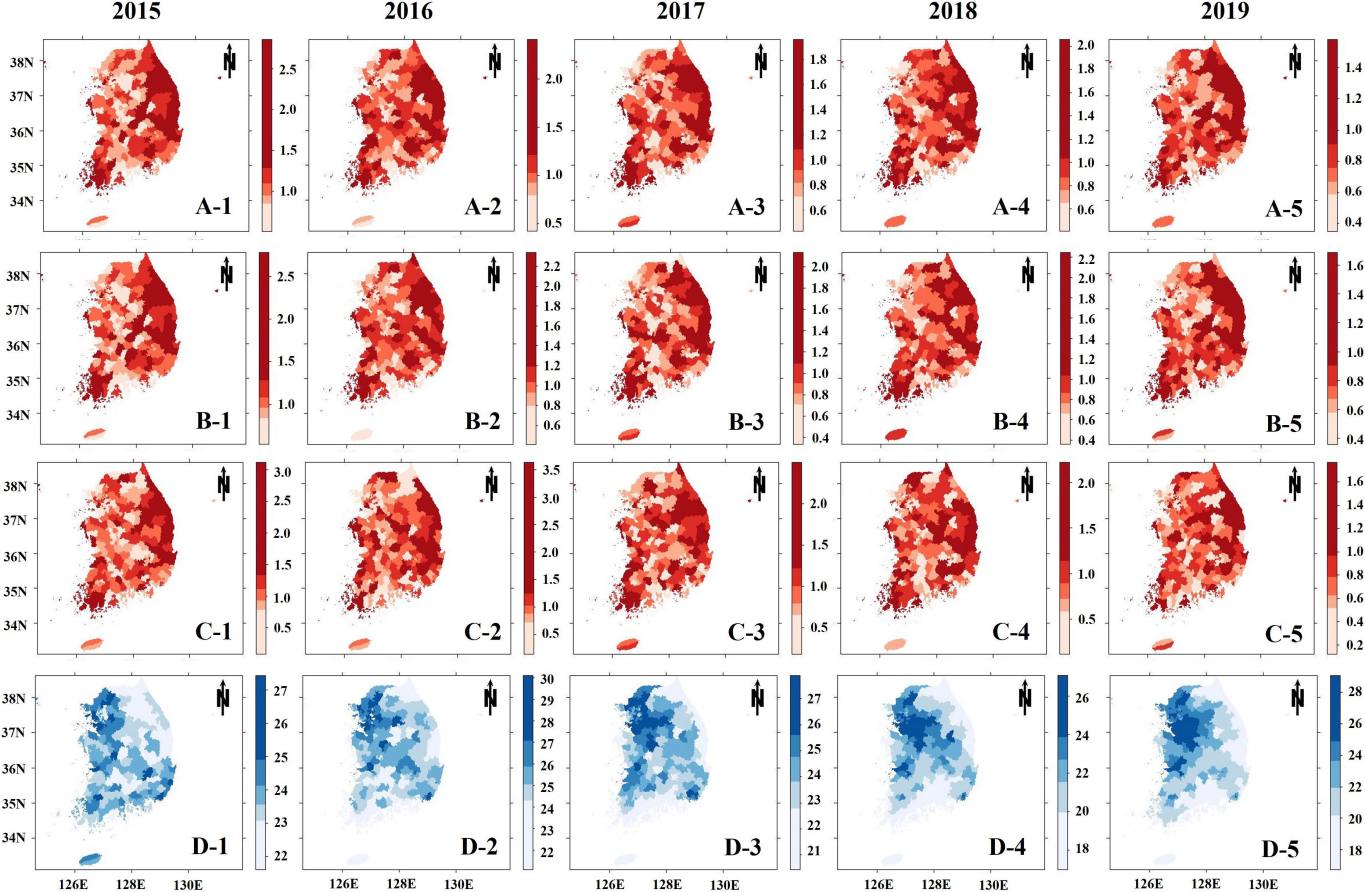
Geographical distribution of standardized tuberculosis incidence ratio and predicted PM_2.5_ concentrations *Note*: A, B, C indicate standardized all, new, and relapsed tuberculosis incidence ratio, respectively and D represents predicted PM_2.5_ concentrations between 2015 (left, 1) and 2019 (right, 5) by quintile classifications

Table 1 summarized the general characteristics of 1,250 study subjects (250 districts × 5 years). The districts with a higher all TB SIR tended to have higher prevalence of smoking, binge drinking, and diabetes but have lower population density, quality of life and budget dependency compared with the districts with a lower all TB SIR. There are no significant differences in PM_2.5_ concentrations and obesity prevalence between the districts with higher and those with lower all TB SIR. The univariable associations were similar with new TB SIR (Table S1) and relapsed TB SIR (Table S2). Geographical distribution of standardized TB incidence ratios and PM_2.5_ concentrations were shown in Fig. 1. TB SIR tended to dispersed over country, but eastern part showed relatively higher incidence. On the other hand, predicted PM_2.5_ level was higher in north-western part. However, univariate associations between the predicted PM_2.5_ and TB SIRs were not significant and Pearson’s correlation coefficients were close to 0 (Fig. S1). One-to-one correlations among explanatory variables were illustrated in Fig. S2 to check multi-collinearity. All pairs of variables showed low to moderate correlation (<0.6).

**Table 1:** General characteristics of the study subjects (N = 1250; 250 districts in 5 years)

**Variable**	**Districts with high TB SIR^1^** **(n = 625)**	**Districts with low TB SIR^1^** **(n = 625)**	***p*-value** **(*t*-test)**
PM_2.5_ (annual mean, µg/m^3^)	23.66 ± 2.4	23.69 ± 2.2	0.816
Smoking prevalence (%)	22.47 ± 2.7	20.71 ± 2.8	<0.0001
Binge drinking prevalence (%)	15.24 ± 3.1	14.41 ± 2.8	<0.0001
Diabetes prevalence (%)	8.19 ± 1.2	7.83 ± 1.3	<0.0001
Obesity prevalence (%)	29.24 ± 4.0	29.19 ± 3.8	0.831
Budget dependency (%)	18.96 ± 13.0	27.18 ± 14.1	<0.0001
Population density (10^3^ per km^2^)	3.18 ± 5.8	4.76 ± 6.1	<0.0001
Quality of life index^2^	95.54 ± 1.0	95.78 ± 0.9	<0.0001

^1^Age- and sex-standardized all tuberculosis incidence ratio

^2^Range for quality of life index is between 0 and 100

*Note*: Categorization of districts by all TB SIR was based on the median value

**Fig. 2:**
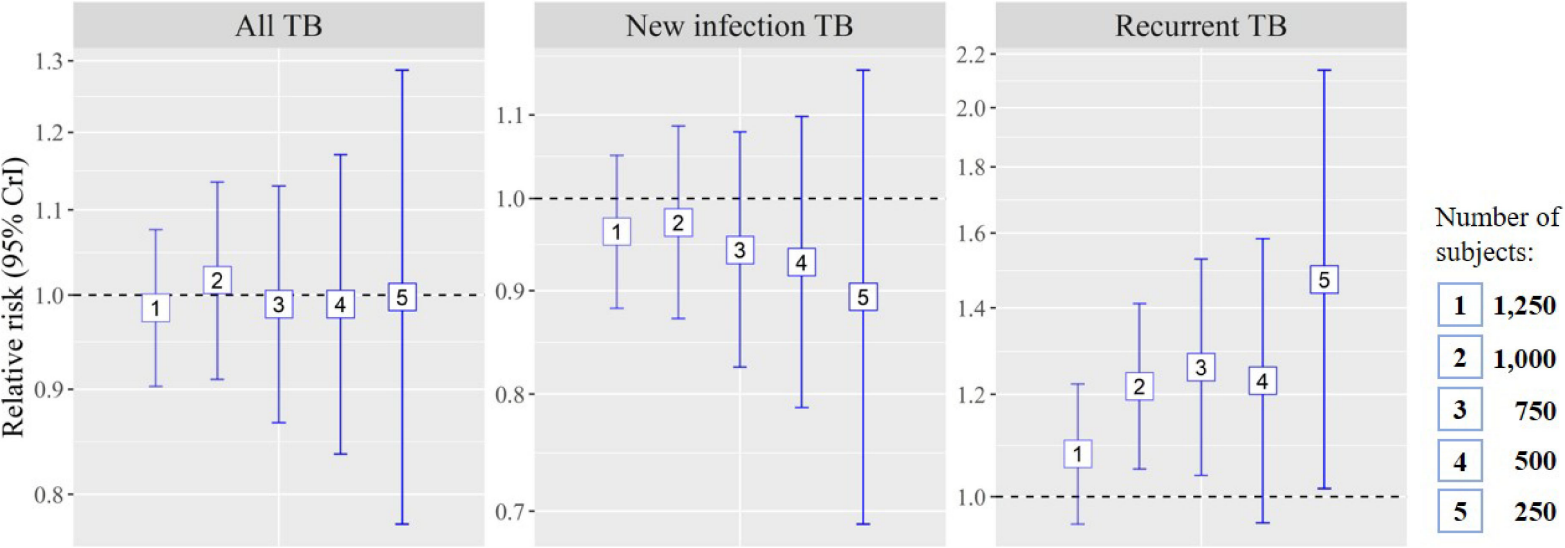
Relative risk for standardized all, new infection and recurrent TB incidence ratio *Note*: The relative risk (RR) was estimated as increased risk per 10 µg/m^3^ PM_2.5_ concentration increase and 95% credible interval (CrI) was also suggested. The RRs for standardized all (left), new infection (middle) and recurrent (right) TB incidence ratio were estimated by negative binomial regression models incorporating spatial autocorrelation. Numbers in the middle of error bars indicate time windows for PM_2.5_ exposures. For example, ‘1’ means one-year average of PM_2.5_ exposures and the error bar indicates the association of TB SIR with PM_2.5_ exposures in the same year. On the other hand, ‘2’ means two-year average of PM_2.5_ exposures and the error bar indicates the association of TB SIR with average PM_2.5_ exposures in the same and previous years. See Methods for further details.

Considering that DIC of NB models were lower than Poisson models in all types of models, NB models were selected as our final models. Figure 2 shows the associations of PM_2.5_ concentrations with all, new and relapsed TB SIR by NB models. Regardless of PM_2.5_ averaging periods, all effect estimates of all and new TB SIR were not significant. However, significant positive associations were found between the two, three and five-year average PM_2.5_ concentrations and relapsed TB SIR, with RRs of 1.218 (95% CrI = 1.051–1.411), 1.260 (95% CrI = 1.039–1.527) and 1.473 (95% CrI = 1.015–2.137), respectively.

**Fig. 3:**
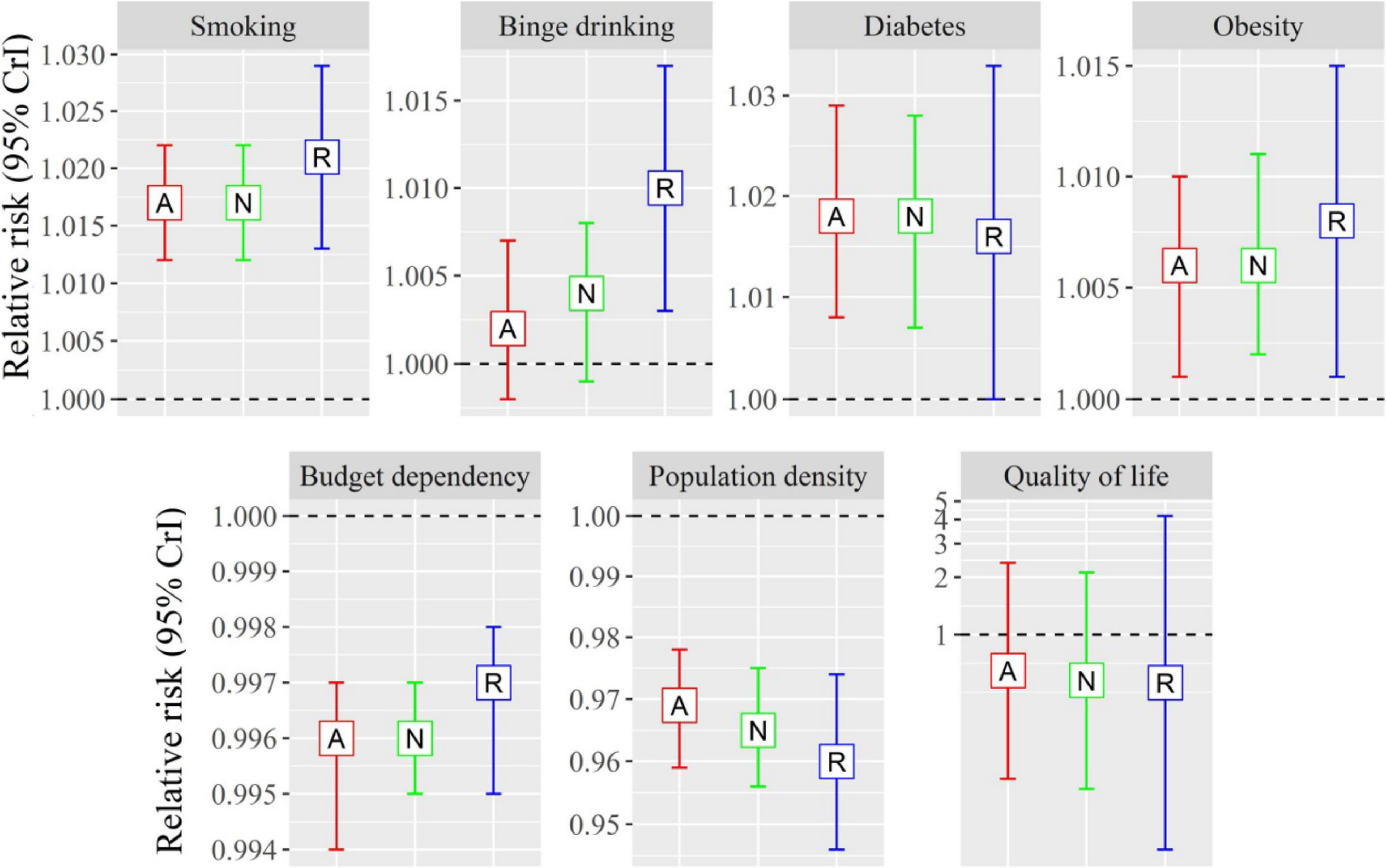
Associations between covariates and standardized all, new infection and recurrent TB incidence ratio *Note*: A, N and R indicate the associations with standardized all, new infection and recurrent TB incidence ratio, respectively. Y-axis indicates relative risk and 95% credible interval (CrI). The units of each variable are presented in Table 1.

Figure 3 represented associations of covariates with all, new and relapsed TB SIR. Regardless of types of TB, prevalence of smoking, obesity and diabetes showed significantly positive associations, but population density and budget dependency showed significantly negative associations. In addition, prevalence of binge drinking was positively associated with relapsed TB SIR.

## Discussion

This study investigated the association between long-term ambient PM_2.5_ exposures and the risk of new and relapse TB in a district-level ecological study. Despite the limited sample size, we found that districts with higher PM_2.5_ exposures for the two, three and five year-average tended to have a higher standardized incidence ratio of recurrent TB. However, we did not find the association with new TB infection.

The significantly positive association between PM_2.5_ exposure and TB recurrence is consistent with previous experimental [8–10] and epidemiological findings [33]. In addition, the longer exposure time window tends to have higher RR, possibly due to long duration between completion of treatment for primary infection and reactivation. A previous study revealed that the duration could be up to 100 months [34]. Cumulative exposure to PM_2.5_ over years could compromise immunological equilibrium [16] and consequently contribute to reactivation. A previous study in South Korea also reported similar findings that longer time window for PM_10_ exposure showed higher effect estimates on total TB notification rate [21].

On the other hand, the null associations of PM_2.5_ exposure with new TB infection were inconsistent with previous studies [11, 12, 15]. There could be two possible explanations. First, the previous studies examined relatively short-term effect of PM_2.5_ while this study focused on the long-term effect. The short-term exposure measures could capture two possible underlying mechanisms in terms of increasing risk of new infection; 1) the exposure could downregulate immune responses, and 2) particulate matter could be a carrier for TB pathogens which increases transmissibility [35]. However, the long-term exposure measures have limited to capture the latter mechanism which in turn could dilute the associations. Second, different models were used. Previous studies with ecological study design often used Poisson regression to examine the association [11, 13, 15, 21]. However, Modelling count data with overdispersion by Poisson regression could exaggerate estimates that lead to false positive findings [36]. Because there were lack of descriptions for examining overdispersion in the previous studies, possibility of exaggerated results cannot be excluded. Our results with Poisson regression also showed positive association between new TB infection and PM_2.5_ exposures (Fig. S3), but results with NB models which were our final models showed null associations. In addition, our models did not adjust important factors, such as contact density and active TB prevalence which might have large effects on new TB infection and have diluted the effect of PM_2.5_ exposures. Further studies are recommended to examine the effect of PM_2.5_ on new TB infection using hybrid approach incorporating both mathematical and statistical models. Using outcome measures as reproductive number rather than number of cases (or incidence rate) could efficiently adjust the contact density or active TB prevalence [37].

Associations of covariates to TB SIRs were generally consistent with previous findings, such as significantly positive associations of smoking, drinking and diabetes prevalence [4] and negative associations of high socio-economic status [5]. The low socio-economic status can be associated with proximate risk factors that affect immune system. For example, people with lower socio-economic status tend to suffer malnutrition, exposure to indoor pollution, depression and stress [5] which were not adjusted in the models. However, the positive associations of obesity prevalence were opposite to previous findings [22, 38]. Considering that low-income group tend to have higher risk of obesity in South Korea [39], the higher obesity prevalence in this study could represent lower socio-economic status. The negative associations of population density can be interpreted in the same aspect, because regional socio-economic status tend to be lower in rural area where population density is also lower than urban area. Although budget dependency was included in the model, the variable represents financial soundness rather than income-level of residents. Further studies with individual-level study design are recommended for better adjustment of socio-economic status.

The limitations of this study should be considered for proper interpretation. First, this study used predicted PM_2.5_ concentrations. Because there are fewer air pollution-monitoring sites in rural areas, the validity of the predicted PM_2.5_ concentrations could be low in rural areas. Second, the individual-level association between PM_2.5_ exposure and TB incidence should be examined considering the ecological fallacy issue from the area-level analysis. Third, we used district-specific data on TB cases obtained from the KDCA, which includes not only pulmonary TB but also other types of TB infection, such as zoonotic TB. However, the majority of TB cases were pulmonary TB [2]. Fourth, relapsed TB cases included treatment failure and incomplete treatment for primary infections. However, the majority (e.g., 85.4% in 2019) of relapsed TB cases experienced complete and successful treatment for primary infections [2].

## Conclusions

In conclusion, we report the significant association between long-term ambient PM_2.5_ exposures and the risk of new and relapse TB in a district-level ecological study. Our results imply that interventions to reduce air pollution would help reduce the risk of recurrent TB. Based on the plausible biological mechanism of immune response, the association with PM_2.5_ in our findings could be applicable to other infectious diseases, such as influenza [40] and coronavirus disease 2019 [41, 42]. Future studies are highly recommended to accumulate scientific evidence for the effect of air pollution in order to advocate policies to reduce the burden of infectious diseases attributable to air pollution.

## Abbreviations

CrIs: credible intervals
DIC: deviance information criterion
INLA: integrated nested Laplace approximation
KDCA: Korea Disease Control and Prevention Agency
KOSIS: Korean Statistical Information Service
NB: negative binomial
OECD: Organization for Economic Cooperation and Development
PM: particulate matter
RR: relative risks
SES: socioeconomic status
SIR: standardized incidence ratio
TB: Tuberculosis

## Supplementary information

**Additional file 1:** **Figure S1.** Univariate associations between standardized incidence ratio of tuberculosis and predicted PM_2.5_ level. **Figure S2.** Correlation matrix for explanatory variables used in this study. **Figure S3.** Relative risk per 10 µg/m^3^ PM_2.5_ concentration increase and 95% credible interval (CrI) for standardized all (left), new infection (middle) and recurrent (right) TB incidence ratio estimated by Poisson regression models incorporating spatial autocorrelation. **Table S1.** General characteristics of the study subjects categorized by standardized incidence ratio of annual new tuberculosis (N = 1,250, 250 districts in 5 years). **Table S2.** General characteristics of the study subjects categorized by standardized incidence ratio of annual recurrent tuberculosis (N = 1,250, 250 districts in 5 years) (N = 1,250, 250 districts in 5 years).

## Declarations

### Availability of data and materials

The datasets used and/or analysed during the current study are available from the corresponding author on reasonable request. In terms of number of TB cases by district, the data is publicly available in the following KCDA web site. (https://www.kdca.go.kr/npt/biz/npp/portal/nppPblctDtaMain.do?pblctDtaSeAt=1)
