# Supplementary material for: Ambient PM2.5 exposures could increase risk of tuberculosis recurrence
Source: Environ Health Prev Med. 2023 Aug 31;28:48. doi: 10.1265/ehpm.23-00131 (PMC10480611; doi:10.1265/ehpm.23-00131)
Supplement: Supplementary file 1 — Additional file 1: Figure S1. Univariate associations between standardized incidence ratio of tuberculosis and predicted PM2.5 level. Figure S2. Correlation matrix for explanatory variables used in this study. Figure S3. Relative risk per 10 µg/m3 PM2.5 concentration increase and 95% credible interval (CrI) for standardized all (left), new infection (middle) and recurrent (right) TB incidence ratio estimated by Poisson regression models incorporating spatial autocorrelation. Table S1. General characteristics of the study subjects categorized by standardized incidence ratio of annual new tuberculosis (N = 1,250, 250 districts in 5 years). Table S2. General characteristics of the study subjects categorized by standardized incidence ratio of annual recurrent tuberculosis (N = 1,250, 250 districts in 5 years) (N = 1,250, 250 districts in 5 years). [file ehpm-28-048-s001.docx]

Supplementary information


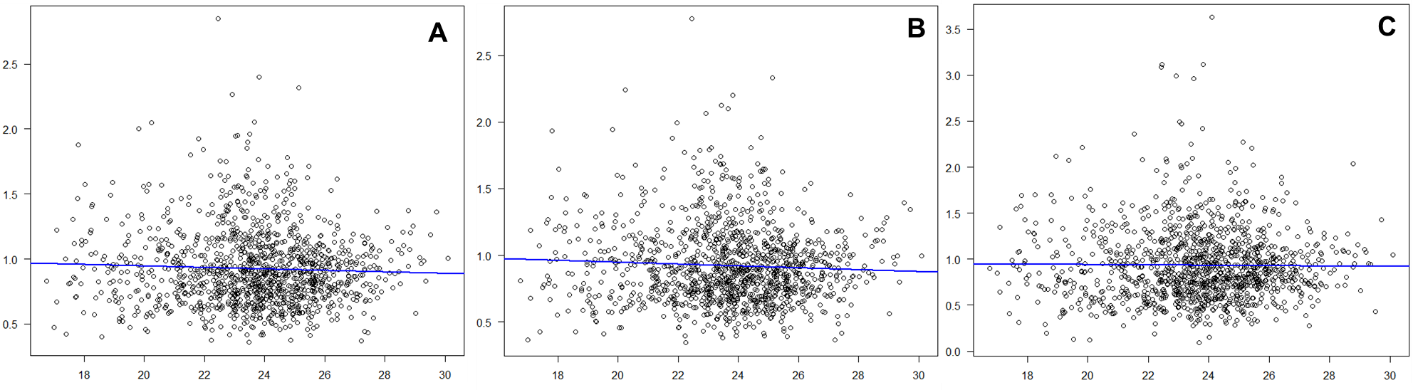


Figure S1. Univariate associations between standardized incidence ratio of tuberculosis and predicted PM_2.5_ level

*Note*: Character A, B, and C indicate the associations of predicted PM_2.5_ levels with all, new and relapsed tuberculosis incidence, respectively. Pearson’s correlation coefficients and *p* value for each association were -0.045 (*p* = 0.111), -0.054 (*p* = 0.056), and -0.009 (*p* = 0.745), respectively.


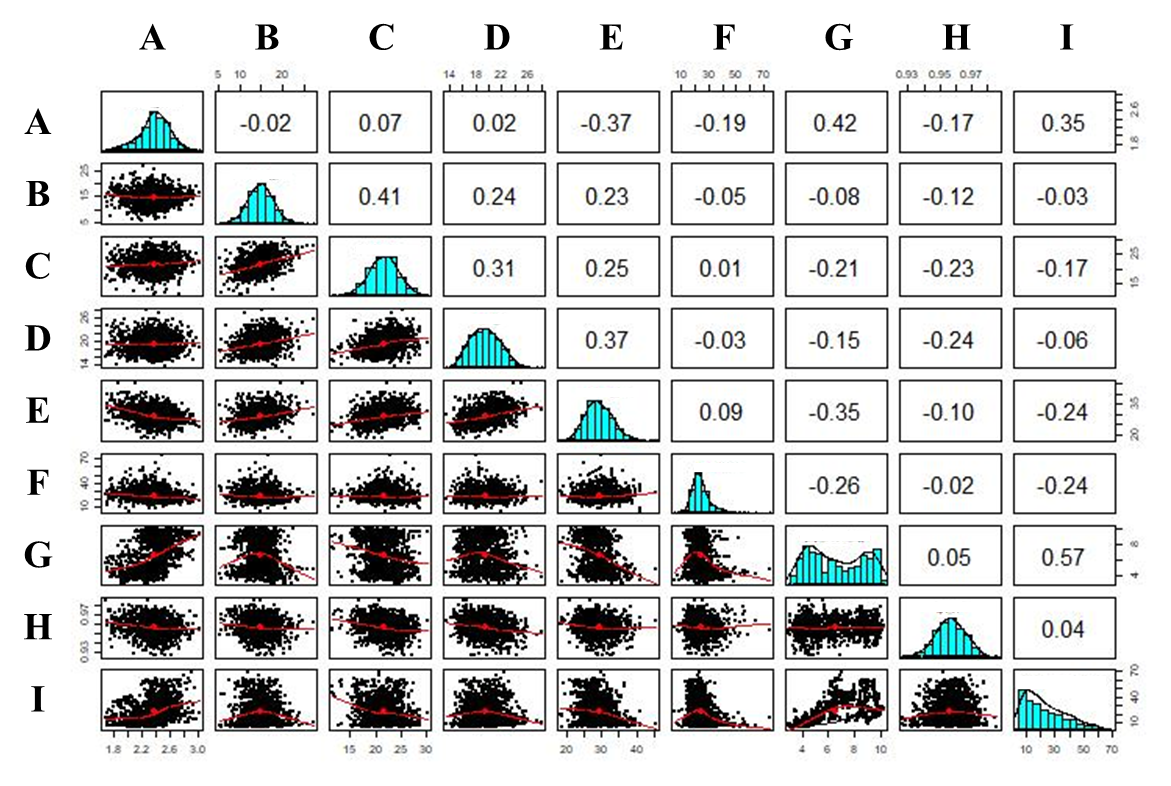


Figure S2. Correlation matrix for explanatory variables used in this study

*Note*: Character A to I indicates predicted PM_2.5_ concentrations, proportion of binge drinkers, proportion of smokers, hypertension prevalence, obesity prevalence, proportion of regular exercisers, population density, quality of life index, and budget dependency, respectively. We assumed there is no multicollinearity because all correlation coefficients were less than 0.6.


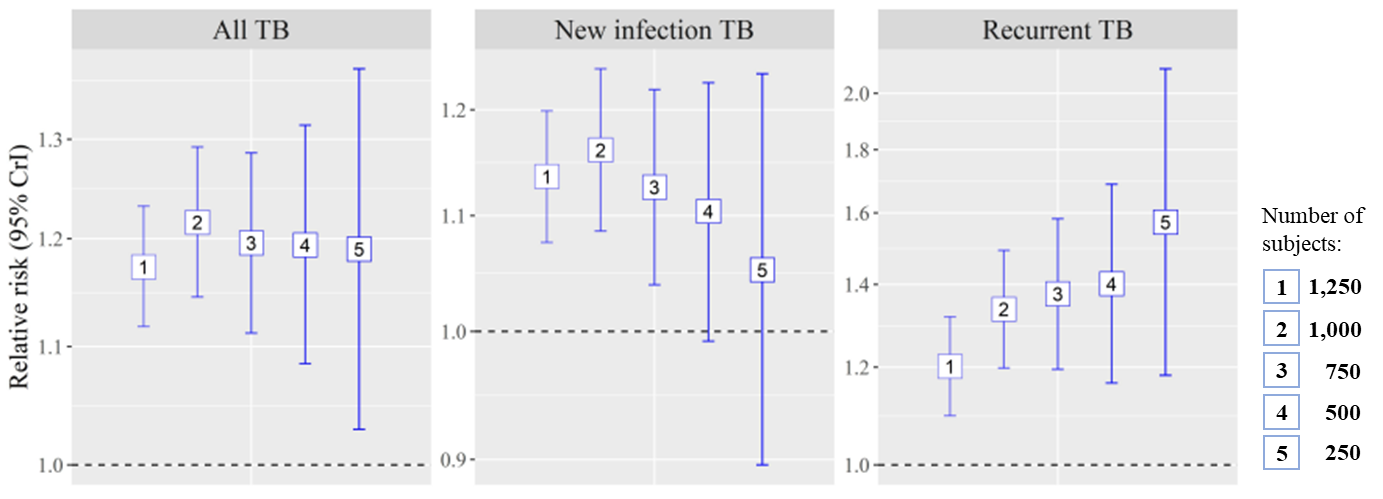


Figure S3. Relative risk per 10 µg/m^3^ PM_2.5_ concentration increase and 95% credible interval (CrI) for standardized all (left), new infection (middle) and recurrent (right) TB incidence ratio estimated by Poisson regression models incorporating spatial autocorrelation

Table S1. General characteristics of the study subjects categorized by standardized incidence ratio of annual new tuberculosis (N=1,250, 250 districts in 5 years)

| Variable | Districts with high TB SIR^1^  (n=625) | Districts with low TB SIR^1^  (n=625) | *p*-value (*t*-test) |
| --- | --- | --- | --- |
| PM_2.5_ (annual mean, µg/m^3^) | 23.64 ± 2.4 | 23.71 ± 2.2 | 0.618 |
| Smoking prevalence (%) | 22.41 ± 2.8 | 20.77 ± 2.8 | <0.0001 |
| Binge drinking prevalence (%) | 15.18 ± 3.1 | 14.47 ± 2.8 | <0.0001 |
| Diabetes prevalence (%) | 8.16 ± 1.3 | 7.86 ± 1.3 | <0.0001 |
| Obesity prevalence (%) | 29.20 ± 4.1 | 29.23 ± 3.7 | 0.891 |
| Budget dependency (%) | 19.15 ± 13.0 | 26.99 ± 14.1 | <0.0001 |
| Population density (10^3^ per km^2^) | 3.18 ± 5.8 | 4.76 ± 6.1 | <0.0001 |
| Quality of life index ^2^ | 95.53 ± 1.0 | 95.78 ± 0.9 | <0.0001 |

^1^Age-and sex- standardized new tuberculosis incidence ratio

^2^ Range for quality of life index is between 0 and 100

*Note*: Categorization of districts by new TB SIR was based on the median value

Table S2. General characteristics of the study subjects categorized by standardized incidence ratio of annual recurrent tuberculosis (N=1,250, 250 districts in 5 years) (N=1,250, 250 districts in 5 years)

| Variable | Districts with high TB SIR^1^  (n=625) | Districts with low TB SIR^1^  (n=625) | *p*-value (*t*-test) |
| --- | --- | --- | --- |
| PM_2.5_ (annual mean, µg/m^3^) | 23.74 ± 2.4 | 23.62 ± 2.2 | 0.356 |
| Smoking prevalence (%) | 22.33 ± 2.7 | 20.85 ± 2.9 | <0.0001 |
| Binge drinking prevalence (%) | 15.25 ± 3.0 | 14.40 ± 2.9 | <0.0001 |
| Diabetes prevalence (%) | 8.14 ± 1.2 | 7.88 ± 1.3 | 0.0004 |
| Obesity prevalence (%) | 29.22 ± 4.0 | 29.20 ± 3.9 | 0.927 |
| Budget dependency (%) | 19.99 ± 13.2 | 26.15 ± 14.4 | <0.0001 |
| Population density (10^3^ per km^2^) | 3.26 ± 5.7 | 4.68 ± 6.2 | <0.0001 |
| Quality of life index ^2^ | 95.55 ± 1.0 | 95.76 ± 0.9 | 0.0001 |

^1^Age-and sex- standardized recurrent tuberculosis incidence ratio

^2^ Range for quality of life index is between 0 and 100

*Note*: Categorization of districts by recurrent TB SIR was based on the median value
